# Transcriptional analysis of susceptible and resistant European corn borer strains and their response to Cry1F protoxin

**DOI:** 10.1186/s12864-015-1751-6

**Published:** 2015-07-29

**Authors:** Neetha Nanoth Vellichirammal, Haichuan Wang, Seong-il Eyun, Etsuko N. Moriyama, Brad S. Coates, Nicholas J. Miller, Blair D. Siegfried

**Affiliations:** Department of Entomology, University of Nebraska-Lincoln, Lincoln, NE USA; Center for Biotechnology, University of Nebraska-Lincoln, Lincoln, NE USA; School of Biological Sciences and Center for Plant Science Innovation, University of Nebraska-Lincoln, Lincoln, NE USA; USDA-ARS, Corn Insects and Crop Genetics Research Unit, Ames, IA USA

**Keywords:** European corn borer, *Ostrinia nubilalis*, Insect resistance, Cry1F resistance, Bt-toxin, Transcriptomics, Cry1F response, RNA-Seq

## Abstract

**Background:**

Despite a number of recent reports of insect resistance to transgenic crops expressing insecticidal toxins from *Bacillus thuringiensis* (Bt), little is known about the mechanism of resistance to these toxins. The purpose of this study is to identify genes associated with the mechanism of Cry1F toxin resistance in European corn borer (*Ostrinia nubilalis* Hübner). For this, we compared the global transcriptomic response of laboratory selected resistant and susceptible *O. nubilalis* strain to Cry1F toxin. We further identified constitutive transcriptional differences between the two strains.

**Results:**

An *O. nubilalis* midgut transcriptome of 36,125 transcripts was assembled *de novo* from 106 million Illumina HiSeq and Roche 454 reads and used as a reference for estimation of differential gene expression analysis. Evaluation of gene expression profiles of midgut tissues from the Cry1F susceptible and resistant strains after toxin exposure identified a suite of genes that responded to the toxin in the susceptible strain (*n* = 1,654), but almost 20-fold fewer in the resistant strain (*n* = 84). A total of 5,455 midgut transcripts showed significant constitutive expression differences between Cry1F susceptible and resistant strains. Transcripts coding for previously identified Cry toxin receptors, cadherin and alkaline phosphatase and proteases were also differentially expressed in the midgut of the susceptible and resistant strains.

**Conclusions:**

Our current study provides a valuable resource for further molecular characterization of Bt resistance and insect response to Cry1F toxin in *O. nubilalis* and other pest species.

**Electronic supplementary material:**

The online version of this article (doi:10.1186/s12864-015-1751-6) contains supplementary material, which is available to authorized users.

## Background

The European corn borer, *Ostrinia nubilalis* Hübner (Lepidoptera: Crambidae), is an economically important pest of corn in the United States and Europe. In the US alone, *O. nubilalis* has been reported to cause over $1 billion in yield losses and control expenditures annually [1]. The introduction of transgenic corn plants expressing *Bacillus thuringiensis* (Bt) crystalline (Cry) toxins in 1996 revolutionized pest management for *O. nubilalis*. Corn hybrids that express Cry1Ab and Cry1F toxins are effective at suppressing *O. nubilalis* feeding damage, and have contributed to recent significant population declines observed throughout the Midwest compared to years prior to 1996 [2]. Grower adoption of Bt-transgenic corn hybrids has been high. These hybrids comprised an estimated 80 % of the US corn crop in 2014 (http://www.ers.usda.gov/data-products/adoption-of-genetically-engineered-crops-in-the-us/recent-trends-in-ge-adoption.aspx). This widespread adoption of Bt-transgenic corn imposes an immense selection pressure on pest insects, leading to the evolution of resistance in the corn pest species *Helicoverpa zea, Spodoptera frugiperda, Busseola fusca* and *Diabrotica virgifera virgifera* (Reviewed in [3]). Cry1F expressing transgenic corn was introduced in 2002 in the US and though resistance to this toxin has not yet been reported in field populations of *O. nubilalis*, resistance has been observed following laboratory selection [4, 5] and resistance alleles have been detected in field populations [6].

*B. thuringiensis* is a gram-positive, spore-forming soil bacterium that produces insecticidal crystal (*Cry*) proteins in insoluble inclusion bodies during the sporulation phase of growth [7]. Individual *Cry* proteins show toxicity toward a subset of arthropod or nematode species, and hence have been deemed safe for mammalian consumption. The generalized mode of action for Bt begins with toxin ingestion and culminates in the death of these insects following disruption of midgut epithelial cells [8, 9]. Two models have been proposed regarding the mechanism by which cell disruption occurs in susceptible lepidopteran species as a result of Bt toxin exposure: 1) the pore formation model and 2) the signal transduction model. According to the pore formation model, toxin monomers bind to receptors on the luminal surface of midgut epithelial cells which leads to toxin oligomerization and insertion into the cell membrane. Embedded toxins are believed to form a pore which affects an ionic balance across the cell membrane and results in cell death due to osmotic lysis (reviewed in [7]). In contrast, the signal transduction model proposes that the binding of the Bt toxin to specific receptors stimulate the G-protein coupled signaling pathway leading to activation of protein kinase A and apoptosis [10]. The specific cell receptors that Cry1F toxins interact with and lead to subsequent toxicity have yet to be identified for *O. nubilalis* [11], although cadherin and aminopeptidases have been implicated based on ligand blot assays [12, 13]. Furthermore, Cry1Ab and Cry1Fa were shown to compete for one or more of the same *O. nubilalis* midgut receptors [14]. Changes in Cry1Ac susceptibility among the laboratory selected *Heliothis virescens* colony YHD2 have been linked to both cadherin and *ABCC* transporter genes, whereas repression of aminopeptidase N transcripts were linked to Cry1Ac and Cry1Ab resistance in *Trichoplusia ni* [15] and *O. nubilalis* [16], respectively.

Understanding how a susceptible and resistant insect respond to sublethal exposure to Cry1F may potentially identify genes associated with the mode of action of this toxin. Moreover, comparing the changes in midgut transcriptomes of Cry1F resistant and susceptible insect strains could detect the differential expression of genes in biochemical pathways associated with Cry1F resistance and provide novel insights into resistance mechanisms in this species. However, the lack of adequate genomic resources has hindered molecular-level studies among lepidopteran pest insects. Transcriptome profiling through massively parallel RNA-Sequencing (RNA-Seq) has transformed research in non-model organisms without prior genomic resources [17, 18]. In this study, we examined the transcriptional differences between a susceptible and resistant *O. nubilalis* strain using the RNA-Seq technology. Due to the lack of reference genome or transcriptome for the *O. nubilalis*, a reference transcriptome was generated by assembly of Roche-454 and Illumina cDNA sequencing data from third instar *O. nubilalis* midgut. In order to examine the molecular mechanisms underpinning the Cry1F toxin response in *O. nubilalis*, we compared transcriptional changes occurring in susceptible and resistant third instars of *O. nubilalis* when exposed to Cry1F protoxin. These strains were selected in the lab for Cry1F resistance and were back-crossed to minimize the genetic background between them. Identifying and comparing transcriptional changes in response to Cry1F protoxin in susceptible and resistant larvae provides unique insight into Cry1F mode of action. In addition, we compared the midgut transcriptional repertoire of third instars of *O. nubilalis* Cry1F susceptible and resistant strains. Analysis of constitutive transcriptional differences between the susceptible and resistant *O. nubilalis* strains provides a global perspective of the evolution of resistance to Bt toxins and the genes that contribute to resistance. Taken together, this approach enhances our understanding pest response to Cry toxin exposure and evolution of Bt resistance.

## Results

### *De novo* assembly, annotation and quality check of the *O. nubilalis* midgut transcriptome

**Fig. 1:**
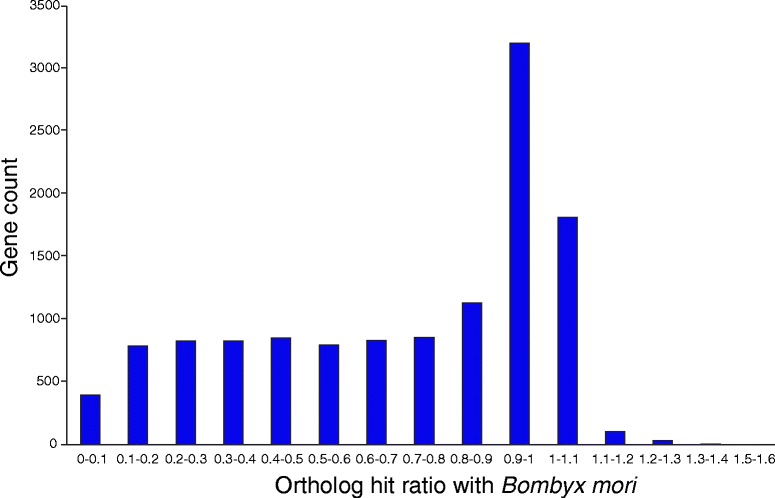
Ortholog hit ratio analysis of the *O. nubilalis* transcriptome assembly. OHR values for the *O. nubilalis* transcripts when compared to *B. mori* sequences. Of the 12,446 *O. nubilalis* sequences with a BLAST hit, 70.42 % have an ortholog hit ratio (OHR) ≥0.5 and 50.49 % have an OHR of ≥0.8

An assembly of 106 million Illumina HiSeq and Roche 454 reads (see Methods section for details) using the short read assembler Trinity yielded 142,083 contigs with an N50 of 1.99 kb and mean length of 1.05 kb including 86,753 unique genes. These contigs were assembled from 501 million reads after filtering and ranged from 201 bases to 24.14 kb in length. After removing redundant sequences and those with low read-counts (less than an average of ten total counts in all samples), the curated assembly consisted of 36,125 transcripts, with an N50 of 2.26 kb (Table 1). The length distribution of these transcripts is shown in Additional file 1: Figure S1. To further assess the quality of the assembled transcriptome, we calculated the ortholog hit ratio (OHR) to estimate the percentage of orthologous genes from *B. mori* contained in each of the assembled *O. nubilalis* transcript. Of the 12,446 *O. nubilalis* contigs that had a BLAST hit to *B. mori* proteins, 6,284 (50.5 %) had OHR > 0.8 and 8,765 (70.4 %) had OHR > 0.5. This indicates that most of the *O. nubilalis* contigs assembled to their full lengths when compared to their orthologs (Fig. 1). A similarity search against the available *O. nubilalis* EST sequences yielded 33.9 % hits, most of which included full length coding regions (67 % had a hit ratio > 0.80). To annotate the assembled transcriptome, a similarity search was done against the protein sequence set from five insect species (*Bombyx mori, Tribolium castaneum, Danaus plexipus, Apis mellifera* and *Drosophila melanogaster*) as well as the non-redundant protein database. In total, 33 % of the *de novo* assembled transcripts yielded BLASTX hits. *O. nubilalis* transcripts had the highest similarities with other lepidopteran sequences including *B. mori* and *D. plexipus*.

**Table 1:** *O. nubilalis denovo* assembly statistics

Number of reads obtained after sequencing	775 million
Number of reads after quality filtering	501 million
Number of assembled transcripts	36,125
N50	2.25 Kb
Mean length of transcripts	1.27 Kb
Minimum transcript length	201 bases
Maximum transcript length	24.12 Kb
No of annotated transcripts	11,967

### Midgut transcriptional repertoire of the susceptible, but not the resistant larvae significantly changes in response to the Cry1F protoxin

**Fig. 2:**
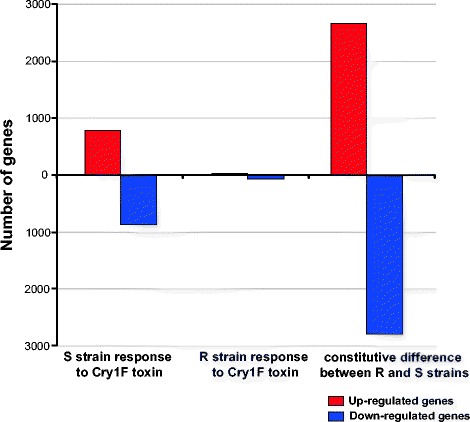
Transcripts that show significant differential expression in the *O. nubilalis* midgut. Results shown for transcriptional response to Cry1F protoxin in the susceptible strain (S), transcriptional response to Cry1F protoxin exposure in the resistant strain (R), and Constitutive differences between the Cry1F resistant and susceptible strains. Cutoffs for estimated differential expression set at FDR adjusted *p*-values ≤ 0.05

In order to understand the differential effects of Cry1F protoxin on midgut transcriptome of susceptible and resistant *O. nubilalis* strains, we estimated transcript quantities before and after Cry1F protoxin exposure using RNA-Seq data. A total of 1,654 transcripts were differentially expressed between the susceptible larvae fed on artificial diet with 500 ng/ml Cry1F and susceptible larvae on artificial diet without Cry1F toxin (Fig. 2). Of these differentially expressed genes, 779 transcripts were upregulated and 875 transcripts were down-regulated in the Cry1F toxin exposed larvae in comparison to the unexposed larvae (Additional file 2: Table S1). Eight transcripts annotated as cytochrome P450 monooxygenases were upregulated in the susceptible strain exposed to Cry1F toxin compared to unexposed controls. Genes encoding carboxypeptidases, aminopeptidase N3, an *ABCC2* transporter, and transcripts involved in ion channel activity were down-regulated in the susceptible strain when exposed to Cry1F toxin. In contrast, when the gene expression of Cry1F protoxin exposed resistant larvae was compared against the unexposed resistant larvae, only 84 transcripts were differentially expressed in the resistant strain (Fig. 2). Of this differentially expressed set, 25 genes were upregulated and 59 genes were down-regulated in the toxin exposed resistant larvae compared to the unexposed larvae (Additional file 3: Table S2). Transcripts found to be upregulated in the Cry1F exposed resistant larvae included serine type endopeptidases and transcripts with transporter activity.

Majority of the transcripts up- or down-regulated in response to Cry1F toxin exposure regardless of strain were assumed to comprise of genes associated with response to protein and might not be involved in toxin resistance or mode of action. A total of 26 genes were up-regulated in both the Cry1F treated resistant and susceptible strains when compared to their untreated counterparts (Additional file 4: Table S3). These included transcripts coding for transmembrane transporter activity, serine type endopeptidase activity and oxido-reductase activity. Expression of 60 transcripts was down-regulated in both strains after Cry1F exposure (Table S3), and included genes coding for metallocarboxypeptidases, GTPases, phosphoinositide 3-kinase and protein transport.

We also catalogued the genes that responded differently to Cry1F toxin exposure in the two strains. For the 842 genes that exhibited significant interaction (*P* < 0.05) between strains and Cry1F treatments, we observed three distinct profiles of gene expression (Additional file 5: Figure S2). The first set (profile 1) included genes with reduced expression after Cry1F exposure in the susceptible strain, but slightly increased expression in the resistant strain after exposure to the toxin (Additional file 6: Table S4). Interestingly, expression of seven transcripts coding for cytochrome P450s were significantly down-regulated in the susceptible strain after toxin exposure compared to the unexposed controls. These transcripts also included serine type carboxypeptidase and those involved in ion channel and transmembrane transport. The second set (profile 2) included genes that were actively transcribed after Cry1F exposure in susceptible strain, but did not change their expression in the resistant strain after exposure to the toxin. This list includes transcripts coding for arylphorin precursor, arylphorin, metallocarboxypeptidase and cysteine-type endopeptidase. A third set of genes (profile 3) did not change their expression in the susceptible strain after toxin exposure, but exhibited slightly increased expression in the resistant strain after exposure to the toxin and included genes involved in lipid and chitin metabolism and transmembrane transport.

### The midgut transcriptional landscape of the resistant larvae differs from the susceptible larvae

**Fig. 3:**
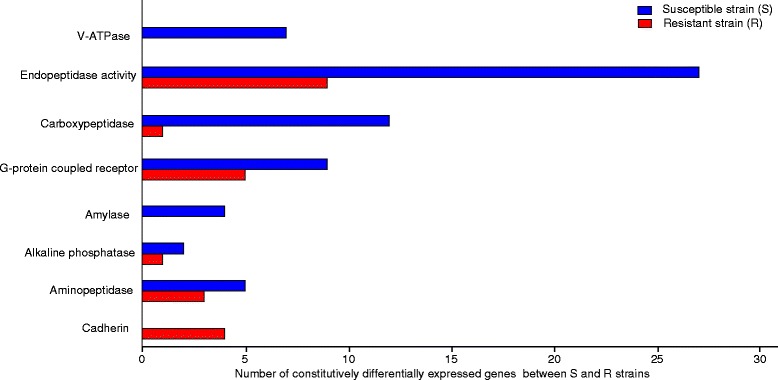
Transcripts differentially expressed between the Cry1F resistant and susceptible *O. nubilalis* strains. The x-axis indicates the total numbers of genes differentially expressed in each category. Only the constitutive differences between the Cry1F resistant and susceptible strains are shown in the figure

We examined the genes that showed constitutive differences between the Cry1F resistant and susceptible strains on normal diet treatments. Even though we backcrossed the resistant and susceptible strains in order to minimize genetic differences, a large number of constitutively differentially expressed genes were detected. Specifically, 2,660 transcripts were up-regulated and 2,795 transcripts were down-regulated in the resistant strain compared to the susceptible strain (Fig. 2 and Additional file 7: Table S5). Manual inspection of these differentially expressed genes revealed transcripts reported to be involved in Bt resistance and mode of action in other species including cadherins, alkaline phosphatases, aminopeptidases, amylases, flotillins, chymotrypsins, cathepsins, G protein coupled receptors, superoxide dismutases, acetyl cholinesterases, carboxylesterases, and endopeptidase activity (Fig. 3). Cadherins, alkaline phosphatases and aminopeptidases have been reported as receptors for Cry toxins (reviewed in [19]). Our data show that four cadherin genes were upregulated in the resistant strain compared to the susceptible strain. The Cry1F susceptible *O. nubilalis* strain expressed more alkaline phosphatase and aminopeptidase genes than the resistant strain. The resistant strain had higher expression of cathepsins, cytochrome P450s and carboxylesterases. In contrast, V-ATPase, genes associated with proteolysis (endopeptidase activity, serine protease, chymotrypsin) and G- protein coupled receptor expression were down-regulated in the resistant strain compared to the susceptible strain. Most of these changes were relatively small with a fold change ranging from 1.4 to 10.6.

### A subset of transcripts in the susceptible strain after Cry1F exposure shifts its expression to resemble the expression in the resistant strain

In this study, we first compared the transcriptional profile of the resistant and susceptible strain to identify the constitutively expressed genes that were differentially expressed. We next identified genes that responded to Cry1F exposure in the susceptible strain. Furthermore, common transcripts that were differentially expressed in both comparisons were identified (Additional file 8: Figure S3). This set included 101 genes that were up-regulated in the resistant strain compared to the susceptible strain and also were up-regulated in the susceptible strain after Cry1F exposure (Additional file 9: Table S6). Transcripts coding for cytochrome P450, potassium channel and transmembrane transport were included in this group. Nearly 200 genes found to be down-regulated in the susceptible strain when compared to the resistant strain and also were found to be repressed in the susceptible strain after Cry1F exposure. These genes included lipases, carboxypeptidases, aminopeptidase N, and V-ATPase subunits (Additional file 10: Table S7).

### qRT-PCR validation

Eight differentially expressed transcripts identified by the different comparisons from RNA-Seq analysis were selected based on their fold change for independent validation of gene expression using quantitative real-time PCR (qRT-PCR). Expression profiles determined by RNA-Seq analysis were consistent with the pattern determined by qRT-PCR, confirming results of the RNA-seq analysis (Table 2).

**Table 2:** Comparison of RNA-seq and qRT-PCR results

Transcript	Comparison	RNA seq Fold change	RT-PCR Fold change
comp30419_c0_seq3	Susceptible Vs Resistant	89.6x up in susceptible	92x up in susceptible
comp55172_c0_seq1	Susceptible Vs Resistant	311.2x up in resistant	16x up in resistant
comp4659_c0_seq1	Susceptible Vs Resistant	10x up in susceptible	8x up in susceptible
comp53943_c0_seq1	Susceptible Vs Resistant	248x up in susceptible	98x up in susceptible
comp32281_c0_seq1	Susceptible Vs Bt treated	116.3x up in susceptible	90x up in susceptible
comp52754_c0_seq1	Susceptible Vs Bt treated	59.5x up in susceptible	90x up in susceptible
comp28866_c0_seq1	Susceptible Vs Bt treated	33.1x up in Bt treated	75x up in Bt treated
comp29067_c0_seq1	Susceptible Vs Bt treated	33.2x up in Bt treated	85x up in Bt treated

## Discussion

In this study, we exposed the Cry1F resistant and susceptible *O. nubilalis* strains to sublethal levels of Cry1F protoxin to analyze the midgut transcriptional response. Our results indicate that the resistant *O. nubilalis* strain generally does not exhibit a strong transcriptional response to Cry1F protoxin exposure. In contrast, the susceptible strain exhibited substantial changes in midgut gene expression in response to this toxin. Specifically, 875 transcripts were down-regulated in the susceptible strain after toxin exposure, and importantly, genes putatively involved in Bt toxin mode of action, such as aminopeptidase N3 (*apn*3), an *ABCC2* transporter and several serine proteases were identified. Such responses may represent the first line of host defense against the toxin to prevent further tissue damage. We identified five transcripts (two coding for trypsin-like serine proteases and three coding for chymotrypsin- like serine proteases) that were down-regulated after Cry1F exposure in the susceptible strain. Yao et al.[20] reported that *O. nubilalis* responds to Cry1Ab protoxin treatment by regulating its protease expression, and suggested that these enzymes might provide a possible mechanism of defense against Cry toxins. Transcripts involved in lipid metabolism were also found to be associated with toxin response. A total of 13 lipases were down-regulated after toxin exposure in the susceptible strain, suggesting that toxins also alter some aspects of lipid metabolism. Collectively, our results indicate a complex response to Cry1F protoxin in *O. nubilalis*, involving regulation of proteases, detoxification enzymes and metabolism.

We also identified several cytochrome P450 transcripts that were differentially expressed after toxin exposure in the susceptible strain. Six cytochrome P450 genes were found to be down-regulated and eight cytochrome P450 genes were upregulated in the toxin treated larvae. These monooxygeneases play an important role in the degradation of insecticides [21] and were also observed to respond to Cry1Ab protoxin in Lepidoptera (*C. fumiferana* and *M. sexta)* [22]. The precise role of cytochrome P450 in Cry protoxin processing is uncertain as these enzymes are generally thought to be involved in biotransformation of lipophilic xenobiotics [23]. However, they also function to regulate titers of many endogenous compounds [21, 24] involved in other pathways that may indicate general response to environmental stress (e.g., Cry1F toxin exposure).

In contrast to results with susceptible larvae, gene expression in the midgut of Cry1F resistant larvae changed very little in response to Cry1F toxin in agreement with very high levels of resistance associated with this strain. This suggests that the toxin fails to engage in the typical mode of action or is incapable of inflicting cellular damage in the midgut of resistant larvae.

Our current understanding of Bt toxin resistance in insects is generally associated with either altered binding of toxins to midgut receptors [25–27] or differences in post binding proteolytic processing of the toxin by the receptors [28, 29]. Decreased binding of the toxins to its midgut receptors in a resistant strain might be due to the structural changes in the receptors or due to decreased expression of these specific receptors in the midgut. Known Cry toxin receptors in Lepidoptera include cadherins [30, 31], glycosylphophatidylinositol (GPI) anchored aminopeptidase N and alkaline phosphatases [32–35]. The majority of the documented molecular mechanisms of Bt resistance in insects are due to mutations of toxin receptor genes [36–39]. In this study, we found four upregulated cadherin transcripts in resistant *O. nubilalis* larvae when compared to the susceptible larvae. Out of these four, two (comp25570_c0_seq1 and comp26081_c0_seq4) had sequence similarity to mutant *Helicoverpa armigera* cadherin (Genbank ID: ACY69027.1) associated with resistance to Cry1Ac. In contrast, other reported Cry toxin receptors such as aminopeptidase N, amylase and alkaline phosphatase were down-regulated in the resistant relative to the susceptible strain. Isoforms of aminopeptidase have been shown to bind to Cry1F toxins in *O. nubilalis* [12]. Our results show that the expression of comp49786_c1_seq1 coding for an aminopeptidase N3 was repressed in the susceptible strain when exposed to Cry1F toxin, indicating that this transcript might be involved in toxin response and also in developing toxin resistance. Similar reduction in aminopeptidase N expression has been associated with Cry1Ac resistance in cabbage looper [15] and Cry1Ab resistance in *O. nubilalis* [16] indicating that this could be a common mechanism of Cry toxin resistance among Lepidoptera.

The Cry1F resistant strain expressed seven transcripts coding for V-ATPase subunits at much lower levels when compared to the susceptible strain. Moreover, when the susceptible strain was Cry1F toxin challenged, expression of four transcripts coding for V-ATPase subunits were significantly reduced in response to toxin exposure. Two of these four transcripts, comp42022_c0_seq1 (coding for subunit E) and comp49953_c1_seq1 (coding for V0 domain) were also expressed at lower levels in the resistant strain. A number of V-ATPase subunits have been reported to bind to different Cry proteins, including Cry1Ab, Cry4Ba and Cry1Ac [40–42] although a role for V-ATPase as a Bt toxin receptor is not yet confirmed. However, V-ATPase subunits have been shown to be involved in sensing and maintaining pH in the insect midgut [43, 44], and are critical to maintaining alkaline conditions of the midgut [45]. Therefore, reduced expression of genes coding for V-ATPase subunits could result in a more acidic midgut in the resistant strain. The pH of the larval midgut is known to affect Cry toxin activity, including solubilization of the crystalline protoxins, protease activity of the midgut and pore formation. As a consequence, alterations in the midgut pH might result in changes in susceptibility. Altered midgut pH and reduced protease activity have been associated with resistance to Cry1Ac and Cry2A protoxins in *H. virescens* [46]. Interestingly, we also observed a number of genes coding for proteases to be down-regulated in the resistant strain compared to the susceptible strain (Fig. 3). Most of these upregulated proteases in the susceptible strain belonged to serine-type endopeptidases which are active at neutral to alkaline pH. On the other hand, proteases upregulated in the resistant strain belonged to aspartic- or cysteine- type endopeptidases with their optimal activities at acidic pH. Examining the role of midgut pH in *O. nubilalis* resistance to Cry1F may provide further insight into potential resistance mechanisms.

There are some reports that have used similar transcriptome sequencing experiments to investigate the mechanism of Bt resistance in other insect species [47–49]. Although different species and Bt toxins were examined, some similarities exist between our observations and these other reports. V-ATPase and alkaline phosphatase were associated with Bt susceptibility in *O. furnacalis and Aedes aegypti* [47, 49] and in the present investigation. Genes associated with proteolytic activity seems to be upregulated in the susceptible strain in at least three of the four reports including the current study [48, 49]. Cadherin receptors were found to be upregulated in the resistant strain of *Aedes aegypti* when compared to the susceptible strain in one other report along with the current study [49]. Though such studies are limited, it is interesting to note common transcripts associated with Bt resistance.

Based on our observations, Cry1F resistance in *O. nubilalis* may involve a combination of several factors although an earlier report identified a single quantitative trait locus associated with Cry1F resistance in *O. nubilalis* that is not linked to any known Bt receptors conferring resistance [50]. This identified QTL is ~19.1 cM in length and is likely to contain more than one gene or encode a transcription factor that can act as a master controller of resistance associated genes. In addition, there is a possibility that several variants of Cry toxin receptors exist rather than a single gene. Therefore, the global transcriptomic analysis described in this report may be more appropriate to capture these genes.

## Conclusion

In this study, we report the first midgut transcriptome for *O. nubilalis*, an economically important pest of corn in the US in which 36,125 transcripts were assembled from the midgut, a target organ for Bt toxin in host insects. We identified a large number of transcripts differentially expressed in Cry1F exposed susceptible *O. nubilalis* strain, with gene functions involved in Bt toxin binding, detoxification and proteolysis. Genes associated with Bt toxin binding, V-ATPase and proteolysis were differentially expressed between the Cry1F resistant and susceptible strains. Results from this study provide key information on the possible molecular mechanisms underlying Cry1F resistance and will serve as a foundation for future investigations into pathways underlying host response to Bt toxins and the evolution of resistance in this and related pest species.

## Methods

### European corn borer rearing and cDNA sequencing

A strain of *O. nubilalis*, BENY (2BE) that was originally collected from a field near Geneva, NY in 1985 was used as a susceptible strain. The Cry1F-resistant strain originated from adult *O. nubilalis* collected throughout the United States Corn Belt in 1996 (115 females and 135 males). Selection for Cry1F resistance was initiated in 1998 [5]. Insects were selected with increasing concentrations of Cry1F incorporated into rearing diet for 30 generations, and maintained at 35 μg Cry1F per ml of diet for ten generations. Further selection was accomplished in 2001 and 2002 using Cry1F applied to the surface of artificial diet and exposing neonates for seven days, after which surviving neonates were transferred to untreated diet. Maintenance of the Cry1F-selected strain was accomplished by exposing neonates to 60 ng Cry1F per cm^2^, corresponding to the upper limit of the 95 % confidence interval of the LC_99_ for susceptible populations, every three generations [5]. This strain displayed a resistance ratio of >3000-fold to Cry1F on diet assays [4, 5]. We repeatedly backcrossed the resistant strain to the susceptible strain, followed by selection on Cry1F, resulting in strains with a common genetic background. Specifically, the selected strain was back-crossed to the susceptible strain, allowed to mate at random for an additional generation, and then selected by rearing on artificial diet with Cry1F applied to the surface of the diet. This process of crossing and selecting was repeated five times. The susceptible strain was maintained simultaneously (and separately). The resulting resistant and susceptible strains were used for the downstream RNA sequencing experiments described in this manuscript. Third instars from the susceptible or resistant strain were exposed to Cry1F protoxin incorporated into the artificial diet [51] at a concentration of 500 ng/ml for 48 h. Third instars were selected for the toxin exposure because they are able to tolerate higher levels of Cry1F protoxin exposure. After the exposure, midguts of larvae were dissected, flash frozen in liquid nitrogen and stored at −80 °C till further processing. Four larval midguts were pooled to process each sample and three replicates were collected per experiment. From each sample, RNA was isolated using the Qiagen RNA isolation kit according to manufacturer’s instructions. The quality of the isolated RNA was analyzed using Bioanalyzer 2100 (Agilent Technologies). From the experimental samples, cDNA libraries were prepared and sequenced on Illumina HiSeq2500 system at the University of Nebraska Medical Center to generate 100 base single end reads. A total of 669 million short reads were generated from sequencing these libraries. Additional Illumina and 454 reads (a total of 106 million reads) were obtained from sequencing a cDNA library prepared from midgut of *O. nubilalis* strain resistant to Cry1F [4], strain resistant to Cry1Ab [52]) and a strain susceptible to both Cry1Ab and Cry1F [5]) were also used for constructing the transcriptome assembly.

### Transcriptome assembly and curation

Pooled sequencing data from both Illumina and 454 sequencing consisted of approximately 775 million reads. The quality of the sequencing reads was analyzed using FastQC v0.10.1 [53]. 454 reads were further processed to remove sequences with more than 1 % N and trimmed to remove adapters and low quality reads with PRINSEQ-lite v0.20.3 [54]. Illumina reads were trimmed to remove adapters and low quality bases using the software Sickle v1.2 (github.com/najoshi/sickle). The resulting 501 million reads were assembled with the Trinity v. r2013-11-10 [55] with default settings (fixed *k*-mer size 25). Sequence redundancy in this *de novo* assembly was removed by searching similar sequences with minimum similarity cut-off of 90 % using CD-HIT-EST [56]. Reads obtained from the experimental samples were aligned against the transcripts with Bowtie v.1.0 [57] with the parameters -qS -n 2 -e 99999999 -l 25 -p 16 -a -m 200 --sam --phred33-quals. Aligned reads from all the samples were further processed to pool sequences representing possible isoforms. Also, contigs with low read counts (average read count below ten across all samples) were removed from further analysis.

### Assessing the quality of the assembly

The quality of the *de novo* assembled transcriptome was assessed using standard metrics including N50 and contig length. In addition, we also calculated the ortholog hit ratio (OHR), which provides an indication of the completeness of a transcript in the assembly [58]. The best BLASTX hit of a transcript against a closely related species was considered to be its ortholog. OHR compares the BLAST hit region in the assembled transcript to the total length of the ortholog [58]. An OHR ratio of 1 would indicate that the transcript has assembled to its full length. BLASTX with an E-value threshold of 10^−10^ was used to identify orthologous genes in the *B. mori* protein database for each assembled *O. nubilalis* transcript. In addition, the transcriptome was also aligned against the EST sequences of *O. nubilalis* available from NCBI (downloaded on March 3, 2014) using BLASTN with an E-value threshold of 10^−10^.

### Annotation

The assembled transcripts were further analyzed to identify putative gene descriptions, conserved domains and gene ontology (GO) terms. The assembled transcripts were searched against *T. castaneum*, *B. mori*, *D. plexipus*, *A. mellifera* and *D. melanogaster* proteins in the Uniprot database with an E-value threshold of 10^−10^ using BLASTX. Sequences with no BLAST hits were then searched against NCBI nr database with an E-value threshold of 10^−30^ and classified functionally by identifying GO terms of molecular function, cellular component, and biological process using the software Blast2GO [59].

### Differential expression analysis

**Fig. 4:**
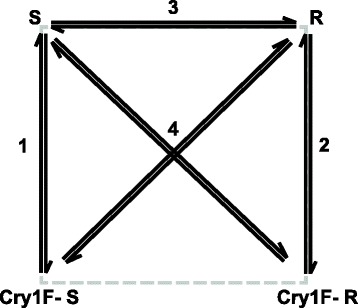
Different gene expression comparisons in this study. 1: Cry1F toxin induced transcriptional differences in the susceptible strain, 2: Cry1F toxin induced transcriptional differences in the resistant strain, 3: Constitutive transcriptional differences between the susceptible and resistant strain, 4: Strain and Cry1F toxin interactions. S and Cry1F-S represents the susceptible and Cry1F treated susceptible strain respectively. R and Cry1F-R represents the resistant and Cry1F treated resistant strain respectively

After pooling the isoforms and filtering transcripts with low read counts, a total of 36,125 transcripts were used for analysis of differential expression. DEseq2 was used to identify differentially expressed (DE) transcripts for all comparisons using default settings [60]. We used a generalized linear model with strain, treatment and interaction term as factors. Transcripts that responded to Cry1F toxin in both strains were identified by comparing the transcriptional profile of Cry1F exposed and unexposed larvae. We identified the transcripts with altered expression between the two *O. nubilalis* strains with different susceptibility to the Cry1F toxin by comparing their midgut larval transcriptomes. The interaction term was used to identify the genes that responded differently to Cry1F exposure in resistant and susceptible strains. Further, we also compared the differentially expressed genes between the two strains and the genes that responded to Cry1F treatment in susceptible strain to identify similar expression profiles. Different comparisons in this study are represented in Fig. 4. Transcripts were considered significantly differentially expressed when corresponding false discovery rate [61] corrected p-values was ≤0.05.

### Validation of differentially expressed genes with qRT-PCR

qRT-PCR was used to validate the differentially expressed gene set obtained from RNA-Seq analysis. Eight genes identified as differentially expressed were selected based on their fold change. Primer pairs were designed using Primer3plus (http://www.bioinformatics.nl/cgi-bin/primer3plus/primer3plus.cgi) and qRT-PCR reactions were performed on the Applied Bio systems 7500 Real-Time PCR System using SYBR Green® (Applied Biosystems) (Additional file 11 Table S8). Three genes [(glyceraldehyde-3-phosphate dehydrogenase (comp15818_c1_seq1), mitochondrial ribosomal protein l28 (comp48444_c2_seq1) and actin (comp11100_c0_seq1)] were selected as reference transcripts based on their gene expression stability across all samples. Out of these three genes, actin was selected as the best reference gene based on the results from Normfinder [62]. Each of the three biological replicates were measured with three technical replicates and fold change was calculated using the 2^−ΔΔCT^ method [63].

### Availability of supporting data

All 454 and Illumina data have been deposited in NCBI’s Sequence Read Archive (SRA) under accession number SRP056082.

## Additional files

Additional file 1: Figure S1. Transcript length distribution of the assembled *O. nubilalis* midgut transcriptome. X-axis is the range of transcript length in bases. Additional file 2: Table S1. Genes differentially expressed in Cry1F protoxin challenged susceptible larvae when compared to control susceptible larvae. Additional file 3: Table S2. Genes differentially expressed in Cry1F protoxin challenged resistant larvae when compared to control resistant larvae. Additional file 4: Table S3. Genes responding to Cry1F protoxin in both susceptible and resistant strains. Additional file 5: Figure S2. Expression profiles of genes interacting with strain and Cry1F toxin exposure. Lane 1: Cry1F untreated susceptible strain 2: Cry1F treated susceptible strain 3: Cry1F untreated resistant strain 4: Cry1F treated resistant strain. Profile 1 includes transcripts that were repressed in Cry1F treated susceptible larvae, but had slightly higher expression in the toxin treated resistant strain. Profile 2 includes transcripts that were upregulated in toxin treated susceptible, but had stable expression in the toxin treated resistant strain. Profile 3 includes transcripts that had stable expression in the toxin treated susceptible strain, but had slightly higher expression in the toxin treated resistant strain. All gene expression values were normalized with that of the Lane 1 (Cry1F untreated susceptible strain). Additional file 6: Table S4. Genes that interacted with strain and toxin response. Additional file 7: Table S5. Genes differentially expressed in the Cry1F resistant strain when compared to the susceptible strain. Additional file 8: Figure S3. Common transcripts differentially expressed between resistant and susceptible strains and between Cry1F toxin response in the susceptible strain. Numbers of up- and down-regulated transcripts are shown from the comparisons between exposed and unexposed susceptible strains (red circle) and between resistant and susceptible strains (green circle). Additional file 9: Table S6. Genes that that were up-regulated in the resistant strain compared to the susceptible strain and also was up-regulated in the susceptible strain after Cry1F exposure. Additional file 10: Table S7. Genes that were down regulated in the susceptible strain when compared to the resistant strain and also was found to be repressed in the susceptible strain after Cry1F exposure. Additional file 11: Table S8. List of primer sequences designed for qRT-PCR.

## References

[1] Mason CE, Rice ME, Calvin DD, Van Duyn JW, Showers WB, Hutchison WD, Witkowski JF, Higgens RA, Onstad DW, Dively GP. European corn borer. Ecology and management. North Central Regional Extension Publication No 327, Iowa State University, Ames, IA 1996.

[2] Hutchison WD, Burkness EC, Mitchell PD, Moon RD, Leslie TW, Fleischer SJ (2010). Areawide suppression of European corn borer with Bt maize reaps savings to non-Bt maize growers. Science.

[3] Tabashnik BE, Brevault T, Carriere Y (2013). Insect resistance to Bt crops: lessons from the first billion acres. Nat Biotechnol.

[4] Pereira EJ, Storer NP, Siegfried BD (2008). Inheritance of Cry1F resistance in laboratory-selected European corn borer and its survival on transgenic corn expressing the Cry1F toxin. Bull Entomol Res.

[5] Pereira EJG, Lang BA, Storer NP, Siegfried BD (2008). Selection for Cry1F resistance in the European corn borer and cross-resistance to other Cry toxins. Entomol Exp Appl.

[6] Siegfried BD, Rangasamy M, Wang H, Spencer T, Haridas CV, Tenhumberg B (2014). Estimating the frequency of Cry1F resistance in field populations of the European corn borer (Lepidoptera: Crambidae). Pest Manag Sci.

[7] Bravo A, Gill SS, Soberon M (2007). Mode of action of Bacillus thuringiensis Cry and Cyt toxins and their potential for insect control. Toxicon.

[8] Rajamohan F, Lee MK, Dean DH (1998). Bacillus thuringiensis insecticidal proteins: molecular mode of action. Prog Nucleic Acid Res Mol Biol.

[9] Schnepf E, Crickmore N, Van Rie J, Lereclus D, Baum J, Feitelson J (1998). Bacillus thuringiensis and its pesticidal crystal proteins. Microbiol Mol Biol Rev.

[10] Zhang X, Candas M, Griko NB, Taussig R, Bulla LA (2006). A mechanism of cell death involving an adenylyl cyclase/PKA signaling pathway is induced by the Cry1Ab toxin of Bacillus thuringiensis. Proc Natl Acad Sci U S A.

[11] Pereira EJ, Siqueira HA, Zhuang M, Storer NP, Siegfried BD (2010). Measurements of Cry1F binding and activity of luminal gut proteases in susceptible and Cry1F resistant Ostrinia nubilalis larvae (Lepidoptera: Crambidae). J Invertebr Pathol.

[12] Hua G, Masson L, Jurat-Fuentes JL, Schwab G, Adang MJ (2001). Binding analyses of Bacillus thuringiensis Cry delta-endotoxins using brush border membrane vesicles of Ostrinia nubilalis. Appl Environ Microbiol.

[13] Tan SY, Cayabyab BF, Alcantara EP, Huang F, He K, Nickerson KW (2013). Comparative binding of Cry1Ab and Cry1F Bacillus thuringiensis toxins to brush border membrane proteins from Ostrinia nubilalis, Ostrinia furnacalis and Diatraea saccharalis (Lepidoptera: Crambidae) midgut tissue. J Invertebr Pathol.

[14] Hernandez-Rodriguez CS, Hernandez-Martinez P, Van Rie J, Escriche B, Ferre J. Shared midgut binding sites for Cry1A.105, Cry1Aa, Cry1Ab, Cry1Ac and Cry1Fa proteins from Bacillus thuringiensis in two important corn pests, Ostrinia nubilalis and Spodoptera frugiperda. PloS one. 2013;8(7):e68164. 10.1371/journal.pone.0068164 PMC3702569 23861865

[15] Tiewsiri K, Wang P (2011). Differential alteration of two aminopeptidases N associated with resistance to Bacillus thuringiensis toxin Cry1Ac in cabbage looper. Proc Natl Acad Sci U S A.

[16] Coates BS, Sumerford DV, Siegfried BD, Hellmich RL, Abel CA (2013). Unlinked genetic loci control the reduced transcription of aminopeptidase N 1 and 3 in the European corn borer and determine tolerance to Bacillus thuringiensis Cry1Ab toxin. Insect Biochem Mol Biol.

[17] Wang Z, Gerstein M, Snyder M (2009). RNA-Seq: a revolutionary tool for transcriptomics. Nat Rev Genet.

[18] Martin JA, Wang Z (2011). Next-generation transcriptome assembly. Nat Rev Genet.

[19] Adang MJ, Crickmore N, Jurat-Fuentes JL (2014). Diversity of Bacillus thuringiensis crystal toxins and mechanism of action., vol. 47.

[20] Yao J, Buschman LL, Oppert B, Khajuria C, Zhu KY (2012). Characterization of cDNAs encoding serine proteases and their transcriptional responses to Cry1Ab protoxin in the gut of Ostrinia nubilalis larvae. PLoS One.

[21] Scott JG, Wen ZM (2001). Cytochrome P450 of insect: the tips of the iceberg. Pest Manag Sci.

[22] van Munster M, Prefontaine G, Meunier L, Elias M, Mazza A, Brousseau R (2007). Altered gene expression in Choristoneura fumiferana and Manduca sexta in response to sublethal intoxication by Bacillus thuringiensis Cry1Ab toxin. Insect Mol Biol.

[23] Hodgson E, Kerkut GA, Gilbert LI (1985). Microsomal monooxygenases. Comprehensive insect physiology, biochemistry and pharmacology vol. 11.

[24] Scott JG. Insect cytochrome P450s: Thinking beyond detoxification. Recent Advances in Insect Physiology, Toxicology and Molecular Biology 2008:117–124

[25] Ferre J, Van Rie J (2002). Biochemistry and genetics of insect resistance to Bacillus thuringiensis. Annu Rev Entomol.

[26] Caccia S, Hernandez-Rodriguez CS, Mahon RJ, Downes S, James W, Bautsoens N (2010). Binding site alteration is responsible for field-isolated resistance to Bacillus thuringiensis Cry2A insecticidal proteins in two Helicoverpa species. PLoS One.

[27] Jurat-Fuentes JL, Karumbaiah L, Jakka SR, Ning C, Liu C, Wu K (2011). Reduced levels of membrane-bound alkaline phosphatase are common to lepidopteran strains resistant to Cry toxins from Bacillus thuringiensis. PLoS One.

[28] Oppert B, Kramer KJ, Beeman RW, Johnson D, McGaughey WH (1997). Proteinase-mediated insect resistance to Bacillus thuringiensis toxins. J Biol Chem.

[29] Zhu YC, Guo Z, Chen MS, Zhu KY, Liu XF, Scheffler B (2011). Major putative pesticide receptors, detoxification enzymes, and transcriptional profile of the midgut of the tobacco budworm, Heliothis virescens (Lepidoptera: Noctuidae). J Invertebr Pathol.

[30] Vadlamudi RK, Weber E, Ji I, Ji TH, Bulla LA (1995). Cloning and expression of a receptor for an insecticidal toxin of Bacillus thuringiensis. J Biol Chem.

[31] Nagamatsu Y, Koike T, Sasaki K, Yoshimoto A, Furukawa Y (1999). The cadherin-like protein is essential to specificity determination and cytotoxic action of the Bacillus thuringiensis insecticidal CryIAa toxin. Febs Lett.

[32] Knight PJ, Crickmore N, Ellar DJ (1994). The receptor for Bacillus thuringiensis CrylA(c) delta-endotoxin in the brush border membrane of the lepidopteran Manduca sexta is aminopeptidase N. Mol Microbiol.

[33] Sangadala S, Walters FS, English LH, Adang MJ (1994). A mixture of Manduca sexta aminopeptidase and phosphatase enhances Bacillus thuringiensis insecticidal CryIA(c) toxin binding and 86Rb(+)-K+ efflux in vitro. J Biol Chem.

[34] McNall RJ, Adang MJ (2003). Identification of novel Bacillus thuringiensis Cry1Ac binding proteins in Manduca sexta midgut through proteomic analysis. Insect Biochem Mol Biol.

[35] Jurat-Fuentes JL, Adang MJ (2004). Characterization of a Cry1Ac-receptor alkaline phosphatase in susceptible and resistant Heliothis virescens larvae. Eur J Biochem.

[36] Gahan LJ, Gould F, Heckel DG (2001). Identification of a gene associated with Bt resistance in Heliothis virescens. Science.

[37] Morin S, Biggs RW, Sisterson MS, Shriver L, Ellers-Kirk C, Higginson D (2003). Three cadherin alleles associated with resistance to Bacillus thuringiensis in pink bollworm. Proc Natl Acad Sci U S A.

[38] Xu X, Yu L, Wu Y (2005). Disruption of a cadherin gene associated with resistance to Cry1Ac {delta}-endotoxin of Bacillus thuringiensis in Helicoverpa armigera. Appl Environ Microbiol.

[39] Fabrick JA, Ponnuraj J, Singh A, Tanwar RK, Unnithan GC, Yelich AJ (2014). Alternative splicing and highly variable cadherin transcripts associated with field-evolved resistance of pink bollworm to bt cotton in India. PLoS One.

[40] Xu L, Ferry N, Wang Z, Zhang J, Edwards MG, Gatehouse AM (2013). A proteomic approach to study the mechanism of tolerance to Bt toxins in Ostrinia furnacalis larvae selected for resistance to Cry1Ab. Transgenic Res.

[41] Bayyareddy K, Andacht TM, Abdullah MA, Adang MJ (2009). Proteomic identification of Bacillus thuringiensis subsp. israelensis toxin Cry4Ba binding proteins in midgut membranes from Aedes (Stegomyia) aegypti Linnaeus (Diptera, Culicidae) larvae. Insect Biochem Mol Biol.

[42] Krishnamoorthy M, Jurat-Fuentes JL, McNall RJ, Andacht T, Adang MJ (2007). Identification of novel CrylAc binding proteins in midgut membranes from Heliothis virescens using proteomic analyses. Insect Biochem Mol Biol.

[43] Onken H, Moffett DF (2009). Revisiting the cellular mechanisms of strong luminal alkalinization in the anterior midgut of larval mosquitoes. J Exp Biol.

[44] Boudko DY, Moroz LL, Linser PJ, Trimarchi JR, Smith PJ, Harvey WR (2001). In situ analysis of pH gradients in mosquito larvae using non-invasive, self-referencing, pH-sensitive microelectrodes. J Exp Biol.

[45] Onken H, Moffett SB, Moffett DF (2008). Alkalinization in the isolated and perfused anterior midgut of the larval mosquito, Aedes aegypti. J Insect Sci.

[46] Karumbaiah L, Oppert B, Jurat-Fuentes JL, Adang MJ (2007). Analysis of midgut proteinases from Bacillus thuringiensis-susceptible and -resistant Heliothis virescens (Lepidoptera: Noctuidae). Comp Biochem Physiol B Biochem Mol Biol.

[47] Xu LN, Wang YQ, Wang ZY, Hu BJ, Ling YH, He KL. Transcriptome differences between Cry1Ab resistant and susceptible strains of Asian corn borer. BMC genomics. 2015;16:173. 10.1186/s12864-015-1362-2 PMC4406038 25886725

[48] Despres L, Stalinski R, Tetreau G, Paris M, Bonin A, Navratil V (2014). Gene expression patterns and sequence polymorphisms associated with mosquito resistance to Bacillus thuringiensis israelensis toxins. BMC Genomics.

[49] Tetreau G, Bayyareddy K, Jones CM, Stalinski R, Riaz MA, Paris M (2012). Larval midgut modifications associated with Bti resistance in the yellow fever mosquito using proteomic and transcriptomic approaches. BMC Genomics.

[50] Coates BS, Sumerford DV, Lopez MD, Wang H, Fraser LM, Kroemer JA (2011). A single major QTL controls expression of larval Cry1F resistance trait in Ostrinia nubilalis (Lepidoptera: Crambidae) and is independent of midgut receptor genes. Genetica.

[51] Siqueira HAA, Moellenbeck D, Spencer T, Siegfried BD (2004). Cross-resistance of CrylAb-selected Ostrinia nubilalis (Lepidoptera : Crambidae) to Bacillus thuringiensis delta-endotoxins. J Econ Entomol.

[52] Crespo AL, Spencer TA, Alves AP, Hellmich RL, Blankenship EE, Magalhaes LC (2009). On-plant survival and inheritance of resistance to Cry1Ab toxin from Bacillus thuringiensis in a field-derived strain of European corn borer, Ostrinia nubilalis. Pest Manag Sci.

[53] Anders S (2010). FastQC a quality-control tool for high-throughput sequence data.

[54] Schmieder R, Edwards R (2011). Quality control and preprocessing of metagenomic datasets. Bioinformatics.

[55] Grabherr MG, Haas BJ, Yassour M, Levin JZ, Thompson DA, Amit I (2011). Full-length transcriptome assembly from RNA-Seq data without a reference genome. Nat Biotechnol.

[56] Li W, Godzik A (2006). Cd-hit: a fast program for clustering and comparing large sets of protein or nucleotide sequences. Bioinformatics.

[57] Langmead B, Trapnell C, Pop M, Salzberg SL (2009). Ultrafast and memory-efficient alignment of short DNA sequences to the human genome. Genome Biol.

[58] O'Neil ST, Dzurisin JD, Carmichael RD, Lobo NF, Emrich SJ, Hellmann JJ (2010). Population-level transcriptome sequencing of nonmodel organisms Erynnis propertius and Papilio zelicaon. BMC Genomics.

[59] Conesa A, Gotz S, Garcia-Gomez JM, Terol J, Talon M, Robles M (2005). Blast2GO: a universal tool for annotation, visualization and analysis in functional genomics research. Bioinformatics.

[60] Love MI, Huber W, Anders S. Moderated estimation of fold change and dispersion for RNA-Seq data with DESeq2. Genome Biol. 2014;15(12):550. 10.1186/s13059-014-0550-8 PMC4302049 25516281

[61] Benjamini Y, Hochberg Y (1995). Controlling the false discovery rate - a practical and powerful approach to multiple testing. J Roy Stat Soc B Met.

[62] Andersen CL, Jensen JL, Orntoft TF (2004). Normalization of real-time quantitative reverse transcription-PCR data: a model-based variance estimation approach to identify genes suited for normalization, applied to bladder and colon cancer data sets. Cancer Res.

[63] Livak KJ, Schmittgen TD (2001). Analysis of relative gene expression data using real-time quantitative PCR and the 2(−Delta Delta C(T)) Method. Methods.

