# Supplementary figures and images for: Transcriptional analysis of susceptible and resistant European corn borer strains and their response to Cry1F protoxin
Source: BMC Genomics. 2015 Jul 29;16(1):558. doi: 10.1186/s12864-015-1751-6 (PMC4518661; doi:10.1186/s12864-015-1751-6)

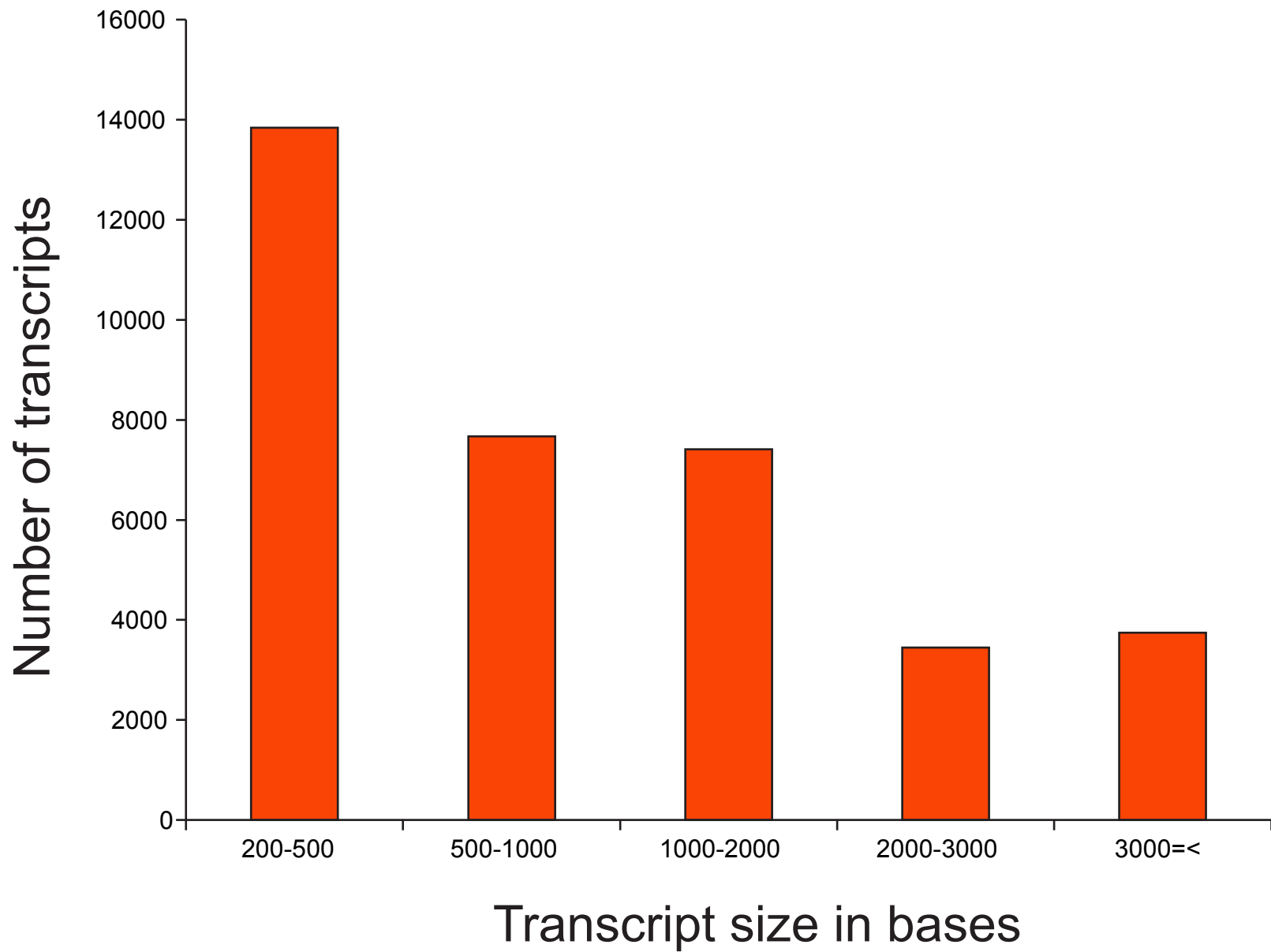

Supplement: Additional file 1: Figure S1. — Transcript length distribution of the assembled O. nubilalis midgut transcriptome. X-axis is the range of transcript length in bases. [file 12864_2015_1751_MOESM1_ESM.pdf]

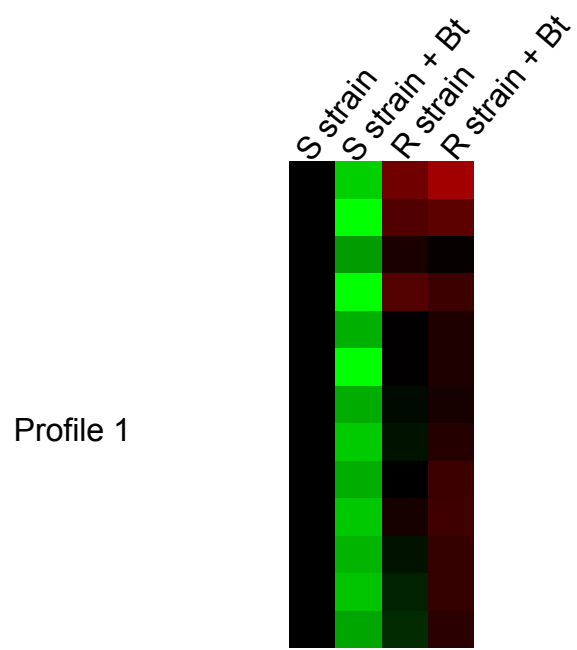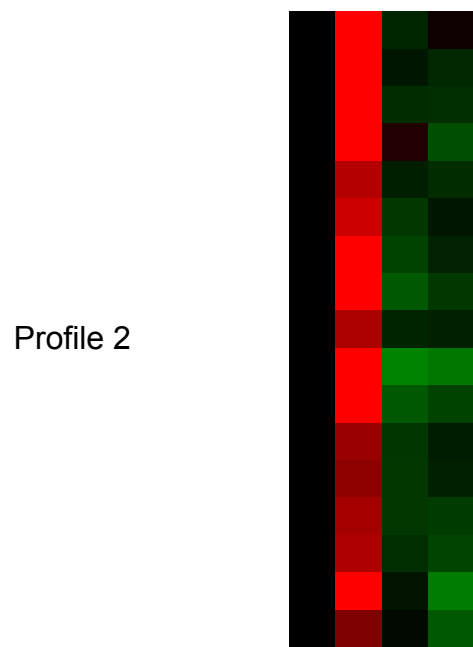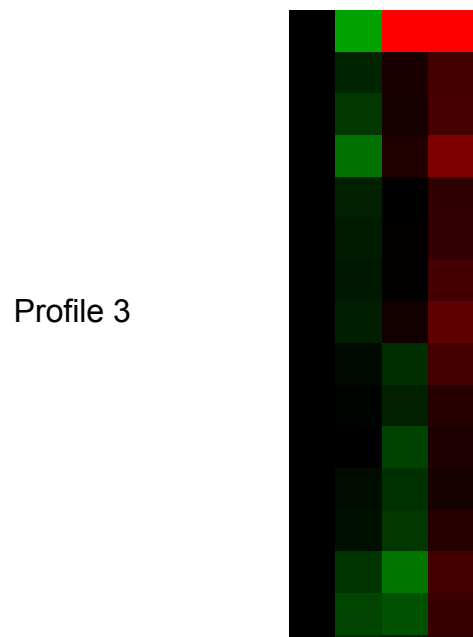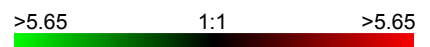

Supplement: Additional file 5: Figure S2. — Expression profiles of genes interacting with strain and Cry1F toxin exposure. Lane 1: Cry1F untreated susceptible strain 2: Cry1F treated susceptible strain 3: Cry1F untreated resistant strain 4: Cry1F treated resistant strain. Profile 1 includes transcripts that were repressed in Cry1F treated susceptible larvae, but had slightly higher expression in the toxin treated resistant strain. Profile 2 includes transcripts that were upregulated in toxin treated susceptible, but had stable expression in the toxin treated resistant strain. Profile 3 includes transcripts that had stable expression in the toxin treated susceptible strain, but had slightly higher expression in the toxin treated resistant strain. All gene expression values were normalized with that of the Lane 1 (Cry1F untreated susceptible strain). [file 12864_2015_1751_MOESM5_ESM.pdf]

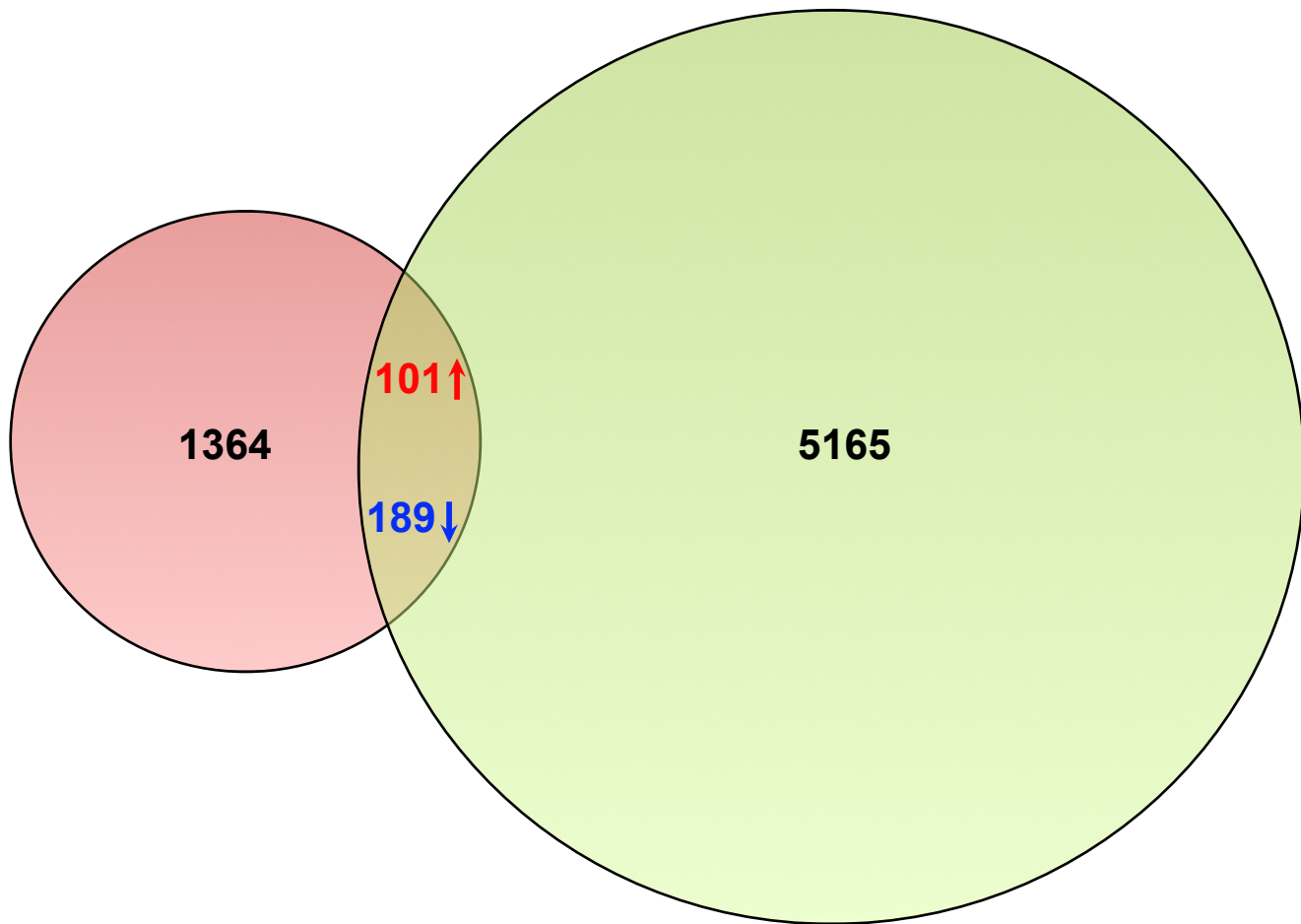

Supplement: Additional file 8: Figure S3. — Common transcripts differentially expressed between resistant and susceptible strains and between Cry1F toxin response in the susceptible strain. Numbers of up- and down-regulated transcripts are shown from the comparisons between exposed and unexposed susceptible strains (red circle) and between resistant and susceptible strains (green circle). [file 12864_2015_1751_MOESM8_ESM.pdf]
